# Practical aspects of lifestyle modifications and behavioural interventions in the treatment of overactive bladder and urgency urinary incontinence

**DOI:** 10.1111/j.1742-1241.2009.02078.x

**Published:** 2009-08

**Authors:** J F Wyman, K L Burgio, D K Newman

**Affiliations:** 1 School of Nursing, University of MinnesotaMinneapolis, MN, USA; 2 Birmingham/Atlanta Geriatric Research, Education and Clinical Center, Department of Veterans Affairs Medical Center and University of Alabama at BirminghamBirmingham, AL, USA; 3 Division of Urology, University of Pennsylvania Health SystemPhiladelphia, PA, USA

## Abstract

Behavioural interventions are effective treatments for overactive bladder (OAB) and urgency urinary incontinence (UUI). They are in part aimed at improving symptoms with patient education on healthy bladder habits and lifestyle modifications, including the establishment of normal voiding intervals, elimination of bladder irritants from the diet, management of fluid intake, weight control, management of bowel regularity and smoking cessation. Behavioural interventions also include specific training techniques aimed at re-establishing normal voiding intervals and continence. Training techniques include bladder training, which includes a progressive voiding schedule together with relaxation and distraction for urgency suppression, and multicomponent behavioural training, which, in conjunction with pelvic floor muscle (PFM) exercises, includes PFM contraction to control urgency and increase the interval between voids. Guidelines for the conservative treatment of OAB and UUI have been published by several organisations and the physiological basis and evidence for the effectiveness of behavioural interventions, including lifestyle modifications, in the treatment of OAB and UUI have been described. However, many primary care clinicians may have a limited awareness of the evidence supporting the often straight-forward treatment recommendations and guidance for incorporating behavioural interventions into busy primary care practices, because most of this information has appeared in the specialty literature. The purpose of this review is to provide an overview of behavioural interventions for OAB and UUI that can be incorporated with minimal time and effort into the treatment armamentarium of all clinicians that care for patients with bladder problems. Practical supporting materials that will facilitate the use of these interventions in the clinic are included; these can be used to help patients understand lifestyle choices and voiding behaviours that may improve function in patients experiencing OAB symptoms and/or UUI as well as promote healthy bladder behaviours and perhaps even prevent future bladder problems. Interventions for stress urinary incontinence are beyond the scope of this review.

**Review Criteria**

The material covered in this review comprises a synthesis of literature gathered from a MEDLINE search based on the terms behavioural intervention, overactive bladder and incontinence, along with the Authors’ clinical experience. Reprints for all articles and book chapters were obtained and assessed for relevance.

**Message for the Clinic**

Behavioural interventions are effective in treating OAB and UUI. Clinicians and staff in busy office settings can readily incorporate these therapies into routine medical and nursing care. Clinicians should be familiar with the practical details of promoting healthy bladder habits, lifestyle modifications and training techniques not only to optimise treatment outcomes, but also as the foundation for patient education and counselling to promote bladder health as part of routine healthcare.

## Introduction

Overactive bladder (OAB), with or without urinary incontinence (UI), is a serious public health problem in the United States and worldwide. OAB is defined by the International Continence Society (ICS) as urgency, with or without urgency incontinence, usually with increased daytime frequency and nocturia (1). Along with urgency urinary incontinence (UUI), which is UI accompanied by urgency, another classification of UI is stress UI, which is defined as UI resulting from effort or exertion, or from sneezing or coughing, and mixed UI, which is a combination of UUI and stress UI (2). It is beyond the scope of this review to address interventions for stress UI; however, the interventions described herein may also provide benefit to these patients.

Overactive bladder is a prevalent condition; the National Overactive BLadder Evaluation (NOBLE) Program found that approximately 16% of men and 17% of women aged ≥ 18 years in the United States are affected by OAB (3). More recently, the EPIC study, which used the current ICS definition of OAB, found that approximately 11% of men and 13% of women in four European countries and Canada reported OAB symptoms (4). Compared with other common chronic diseases, the prevalence of OAB in the United States population is higher than that of diabetes (8%; 2003–2004) (5) and asthma (4.5%; 1988–1994) (6) and comparable to that of eczema (17%) (7). Although the NOBLE and EPIC studies found only slight gender differences in OAB prevalence, UUI is disproportionately more common in women (3). Furthermore, the prevalence of OAB does not differ significantly between ethnic and racial groups in clinic-based populations (8); however, there is some evidence that UUI is more prevalent among black women in population-based studies (9–11).

Overactive bladder becomes more prevalent with age (3,4). This is important because forecasts taken from United Nations statistics predict that the proportion of the population aged 65 years or older will approximately double over the next two decades (12). However, a considerable proportion of the younger population also report OAB symptoms and UUI (4).

Symptoms of OAB with or without UUI are bothersome and are associated with reduced health-related quality of life (3,13,14). The impact of OAB on the lives of both men and women is extensive and includes detrimental effects on social, professional and recreational activities; sexual health and function; and relationships with family members (15–19). The impact of OAB on the social and functional aspects of health-related quality of life may be more severe than that of diabetes (14). OAB with or without UUI is also associated with an increased risk for several comorbidities, including depression, urinary tract infection, skin infections, falls and fractures and vulvovaginitis (3,20–23). Moreover, UUI that can accompany OAB is associated with increased rates of institutionalisation and mortality in older patients (24,25). OAB symptoms and UI may also precipitate adaptive changes in the behaviour of those affected, including prophylactic urination (termed defensive voiding), urination upon first desire, and fluid restriction. Incontinence in general has also been shown to be perceived by women as a barrier to exercise (26).

Options for the treatment of OAB include behavioural interventions, which are collectively considered first-line treatments for OAB symptoms with or without UI. Behavioural interventions are aimed at changing patient lifestyle and behaviour and at teaching techniques to suppress urgency and improve continence skills. Behavioural interventions can be readily prescribed in the primary care setting, either before or concomitantly with pharmacotherapy (27–35). Antimuscarinics, including darifenacin, fesoterodine, oxybutynin, solifenacin, tolterodine and trospium chloride, are pharmacological treatments for OAB. Treatment options available for patients with refractory OAB symptoms include botulinum neurotoxin-A injections into the detrusor muscle; surgical procedures (e.g. augmentation and urinary diversion); neuromodulation, which involves stimulation of the sacral nerves using implanted electrical devices (36); and more recently, posterior tibial nerve stimulation (37). These treatments are beyond the scope of this review and will not be discussed further.

## Behavioural interventions

Recommendations for behavioural interventions in the treatment of OAB and UI have been published by the International Consultation on Incontinence (38), the American College of Obstetricians and Gynecologists (39), the Society of Obstetricians and Gynaecologists of Canada (40), the United Kingdom’s National Institute for Health and Excellence (41) and the United States’ National Institutes of Health (42). Behavioural interventions are well suited to the primary care setting and can be conceptually divided into two categories: the first category includes habits that may be modified to alleviate bladder symptoms or promote bladder health and the second includes training techniques aimed at teaching skills to control the symptoms of bladder dysfunction (Table 1). Healthy bladder habits include lifestyle modifications (eliminating bladder irritants from the diet, managing fluid intake, weight control, managing bowel regularity and smoking cessation) and timed voiding regimens aimed at re-establishing voiding at regular intervals. Patient education regarding normal and abnormal bladder function is helpful in the establishment of healthy bladder habits. Training techniques include bladder training with a progressive voiding schedule using relaxation and distraction techniques for urgency suppression and multicomponent behavioural training, in which patients learn to use pelvic floor muscle (PFM) contraction to control urgency, and ultimately increase the interval between voids, in addition to performing PFM training (PFMT; Table 1).

**Table 1:** Behavioural interventions for managing urinary symptoms and promoting bladder health

		Symptom			
Technique	Description	Frequency	Urgency	UUI	MUI
**Habit changes (managing symptoms and promoting bladder health)**					
Lifestyle modification	Diet, fluid, bowel and weight management; smoking cessation	X	X	X	X
Timed voiding*	Urination at a fixed interval that avoids the symptom (useful for urgency and UI not associated with frequency)		X	X	X
**Training techniques (managing symptoms)**					
Urgency control techniques	Deep breathing and using complex mental tasks (reciting poetry, counting backwards from 100 by 7 s etc.) to ignore urgency	X	X	X	X
Bladder training	Progressively increasing interval between voidings; utilises distraction and relaxation techniques to gradually increase the time between urinations	X	X	X	X
Multicomponent behavioural training*	Teaching to not rush to bathroom in response to urgency and use of PFM contractions to suppress bladder contraction and delay voiding, with use of pelvic floor muscle exercises	X	X	X	X
Pelvic floor muscle training	Daily regimen of pelvic floor muscle contractions to maintain or build strength and endurance			X	X
Delayed voiding*	Progressively increasing interval between onset of urgency and voiding	X	X	X	X

*Using a bladder diary. UUI, urgency urinary incontinence; MUI, mixed urinary incontinence; PFM, pelvic floor muscle.

The evidence supporting the use of these strategies is strongest for behavioural training and bladder training with and without PFMT for the treatment of UUI (30,43–45). Whereas evidence supporting lifestyle modifications is, as yet, relatively limited, there is widespread clinical experience and international expert opinion that support the use of lifestyle modifications for the treatment of OAB and UUI. While most of the literature discusses behavioural interventions in the context of treatment for bladder dysfunctions, information regarding lifestyle modifications and PFMT should be equally as useful in primary care for educating patients in the maintenance of good bladder and PFM function and perhaps even for preventing future bladder problems (46,47).

The educational materials included in Appendix 1 may be useful to both the primary care clinician and the patient as an overview of behaviours to promote bladder health and for the initiation of a behavioural intervention. This focused patient-friendly two-page educational pamphlet developed by Pfizer Inc (New York, NY, USA) gives general information about OAB, lifestyle modifications, PFMT and urgency control strategies (48).

### Healthy bladder habits and lifestyle modifications

#### Patient education and counselling

The foundation of behavioural intervention is patient education to enhance patients’ understanding of normal and abnormal bladder function, which then serves as a basis for their understanding the specific strategies that can be recommended for the prevention and/or management of OAB and UUI. Changing bladder habits, making lifestyle changes and adhering to the training techniques require patients to make significant behavioural changes in their daily activities. It is essential to counsel patients on how to best incorporate these strategies into their lives so that adherence to behavioural interventions, and thus an optimal treatment outcome, is more likely.

A bladder diary which is obtained at the initial evaluation can be a useful tool in counselling patients about their voiding behaviour and patterns (49). Some individuals may be unaware of how often they are voiding and they may be voiding to prevent incontinence rather than responding to a need to empty the bladder. The diary can also be used by patients and clinicians for monitoring treatment progress and efficacy. Typically, a bladder diary is kept for 3–7 days and assesses voiding frequency and timing, the number of incontinent episodes and patients’ fluid intake at initiation of treatment, so that progress can be monitored. Bladder diaries can either be very simple, recording the time of voluntary voiding and UUI episodes, or complex, recording episodes of urgency, severity of urgency, voided volume per micturition and type and amount of fluid intake (Appendix 2). An additional bladder record may be found in the online version of this article (Appendix S1).

#### Timed voiding

Individuals who have urinary urgency and UUI may be unaware of what constitutes an appropriate voiding schedule and may exacerbate their symptoms by changing their voiding pattern. For adults who are cognitively intact, physically capable of self-toileting and capable of keeping bladder diaries, timed voiding is recommended, which involves voiding on a fixed schedule irrespective of the need or desire to urinate (50). Along with re-establishing a healthy voiding schedule, timed voiding is also used as the basis of bladder training (discussed in ‘Bladder training’). Counselling patients that it is not necessary to void with every bladder signal may itself provide new information. Also important is teaching them that the bladder should be emptied every 3–4 h. Habitually ignoring the need to urinate may over-distend the bladder and lead to bladder emptying problems. The interval between urinations will be determined by the patient’s bladder capacity, fluid intake and activity levels as well as environmental factors such as the climatic conditions experienced by the patient and the availability of a bathroom. In clinical practice, it may be difficult to persuade active and busy individuals to comply with a rigid voiding schedule and thus, some flexibility in using this approach may be required.

#### Elimination of bladder irritants from the diet

Some foods and beverages are known to promote diuresis or bladder irritability, which in some people can exacerbate OAB symptoms and UUI. Caffeine in particular has been shown to have a diuretic effect (51) and is a constituent of a variety of beverages and foods. Caffeine-containing products (foods and fluids) may increase OAB symptoms by increasing detrusor pressure (52) and by promoting detrusor muscle excitability (53).

Although the strength of the correlation of caffeine intake with OAB symptoms and UI remains to be resolved, the effects of caffeine are likely dose dependent (54). Patients should therefore be queried for their caffeine intake and advised of adverse effects of caffeine on detrusor overactivity and of the potential benefits of reducing its intake. Patients should also be advised to replace their caffeinated dietary intake with non-caffeinated alternatives and to note any changes in bladder symptoms. If there is no change in bladder symptoms and if patients wish to continue to consume caffeine, they should be advised to restrict this to < 200 mg/day (or two cups of coffee), to decrease urgency and frequency (32). Caffeine is also listed in labels for over 1000 over-the-counter and prescription medications (55).

Other dietary factors that may contribute to OAB symptoms and UUI include carbonated drinks in women (56); there is some anecdotal evidence that eliminating these from the diet may promote continence (32). There is also evidence to suggest that aspartame and other artificial sweeteners induce detrusor contraction in rats (57) and thus may contribute to OAB symptoms.

#### Management of fluid intake

Excessive fluid intake can exacerbate OAB symptoms and incontinence, whereas restriction of fluids may result in an increase in urine concentration that may irritate the bladder mucosa and promote urgency, frequency and urinary tract infections (58). Notably, it is common for clinicians to advise patients to reduce fluid intake to alleviate urinary frequency, emphasising the need to assess the patient’s current fluid intake prior to making any recommendations. An appropriate level of fluid intake is particularly important for older adults, for whom a strong relationship between evening fluid intake and nocturia has been reported (59). The daily volume of fluid intake should be approximately six 8-oz glasses per 24 h (i.e. approximately 1500 ml or 30 ml/kg body weight per 24 h) (60). To reduce nocturia, clinicians often advise patients to reduce fluid intake after 6 pm (or approximately 3–4 h before bedtime) and shift their intake to the morning and afternoon, which anecdotally appears to have good results (61).

#### Management of bowel regularity

Constipation is defined clinically as passing < 3 stools per week; however, from the patient’s perspective, this definition also includes straining while passing a stool (61). In a study of constipation in geriatric hospital patients, the prevalence of constipation was found to be directly correlated to UI (62). Higher rates of constipation have also been found in women and men with OAB than those without OAB (19). Severely constipated women appear to have changes in pelvic floor neurological function (63), including denervation of the external anal sphincter and PFMs (64). Alleviation of constipation has been shown to significantly improve urgency and frequency in older patients (65).

Because chronic constipation is a likely risk factor for OAB and UUI, patients can be advised of lifestyle changes that alleviate the associated straining. These may include self-care practices such as increasing dietary fibre (e.g. wheat bran), moderately increasing fluid intake, engaging in exercise and establishing a routine defecation schedule. Patients should also be advised not to ignore the urge to defecate, but rather to respond promptly to the opportunity to move their bowels.

#### Weight control

Obesity is associated with increased risk for the onset of OAB symptoms (56), and having a body mass index > 30 kg/m^2^ is an independent risk factor for OAB in women (66,67) and UI in older men (68). Obesity has been hypothesised to promote UI by increasing intra-abdominal pressure leading to chronic stress on the pelvic floor that may lead to overt structural damage and neurological dysfunction resulting in UI (69). Surgical weight loss has been shown to reduce UUI in women who are morbidly obese (70). Even moderate weight loss has been shown to improve UI symptoms in overweight women (71–73). Thus, weight loss should be considered as a first-line option for the treatment of UUI in overweight women.

#### Smoking cessation

Research has shown that smoking is strongly associated with lower urinary tract symptoms in men (74–76), with urgency (76) and with UUI in women (77). This association may arise from the increases in intra-abdominal pressure caused by chronic coughing in smokers (77) or from increased detrusor activity, which has been shown to be induced by nicotine in the cat bladder (78). Smoking cessation may result in decreased lower urinary tract symptoms in men (74); however, there is currently no information on whether this is true for women (76). Nevertheless, both men and women who smoke should receive education concerning the relationship between smoking and UI and should be provided with strategies for stopping, although there is currently a lack of evidence that such strategies are effective.

### Training techniques for managing OAB symptoms and UUI

#### Simple urgency control and suppression techniques

In the management of OAB symptoms, patients can be taught to control urgency by performing general relaxation techniques, including slow deep breathing exercises to relax the bladder, decrease the intensity of the urgency and allow the patient to delay voiding and distraction techniques in which patients get involved in tasks that involve mental concentration. Examples include checkbook balancing, Sudoku or crossword puzzles; and using self-motivational statements, such as ‘I can wait’, ‘I can take control’ and ‘I will conquer this’ (79). If the patient is able to isolate and perform a PFM contraction, they can be taught to suppress urgency by performing either a 10-s PFM contraction (80) or five or six rapid and intense PFM contractions (see ‘Multicomponent behavioural training’). These contractions appear to induce their effects by preventing internal sphincter relaxation produced by the micturition reflex, which then results in detrusor relaxation (80). Although strong PFM contractions will be more effective than weak contractions in suppressing urgency, simply making the patient aware of the importance of a well-timed PFM contraction is likely to provide benefit.

#### Bladder training

The objective of bladder training is to restore normal bladder function through the use of a progressive voiding schedule in conjunction with teaching techniques to control and suppress urgency. Bladder training is a suitable treatment for OAB patients with urgency, frequency and all types of UI who are motivated to follow instructions (33,79). Studies using bladder training have reported UI resolution ranging from 12% to 73% and improvement rates ranging from 57% to 87% (81). The technique can be easily administered in an office practice setting where the patient is provided information about normal bladder function and instructions on methods to control urgency discussed above. The patient is initially instructed to follow a timed voiding schedule that typically involves voiding every 30–60 min during waking hours (31,38); these voiding intervals are determined from baseline voiding frequency reported in a bladder diary. The patient is instructed to keep to the schedule regardless of urgency and to use strategies to control urgency if this occurs before the scheduled voiding time. Ideally, the voiding interval should be increased by 15–30 min each week according to the patient’s tolerance to the schedule, until a voiding interval of at least 3–4 h is achieved. Use of a bladder diary is recommended for self-monitoring of progress (79). The urgency suppression techniques described above can be implemented and provide benefit even if it is not feasible for a given patient to follow a rigid voiding schedule.

#### Multicomponent behavioural training

Multicomponent behavioural training is a new response to urgency based on the use of PFM contraction as a critical component to suppress urgency, control incontinence and restore a normal voiding interval. The efficacy of behavioural training alone or in combination with other interventions on both the frequency of UUI episodes and patient-reported outcomes has been established in clinical trials (30,43–45). Reductions in frequency of incontinence range from 60% to 80% (29). One study reported a cure rate of 31% for UI episodes in community-dwelling women receiving a behavioural training regimen (30), which is comparable with those reported for pharmacotherapy with antimuscarinics (35).

The training regimen includes teaching patients how to control the bladder by contracting the striated skeletal PFMs that surround the urethra (29,38). Most patients with UUI report that they rush to the bathroom when they sense the need to void. This type of behavioural training instructs patients to resist the normal tendency to rush because it increases intra-abdominal pressure, and that being near the toilet may actually trigger detrusor contraction. Patients are taught instead to stay still, sit down if possible, contract their PFMs several times to help relax the detrusor, wait for their urgency to subside and only then to walk at a normal pace to the bathroom.

Another component of this intervention is the practice of daily PFM exercises to improve strength and skill. Patients are provided with specific instructions on how to locate their PFM, as well as verbal feedback during a pelvic examination (using vaginal or anal palpation), because 50% of women are unable to achieve this with simple verbal or written instruction alone (82). A useful approach is to ask patients to imagine trying to prevent the passing of gas or pinching off a stool by tightening the ring of muscles around the anus without tensing the muscles of the legs, buttocks or abdomen; this should result in a closing and lifting sensation. Men can be asked to imagine moving the penis up and down without moving any other part of the body. Once the patient is able to isolate the PFM, the daily PFMT regimen includes 45 exercises which are divided into sets of 15 exercises, three times a day. The patient should be encouraged to gradually increase the duration of the contraction to a maximum of 10 s, with an equal period of relaxation between contractions (43). Patients should be advised to practice in various positions including lying, sitting and standing. To promote adherence, clinicians should help patients establish a routine schedule for incorporating the three PFMT sets into their daily lives. For example, the exercises could be scheduled before regular activities such as before meals. Scheduling PFMT at specific times is a more effective strategy than remembering to perform exercise on an *ad hoc* schedule (83). For optimal efficacy, PFMT may need to be continued for 8–12 weeks (35). Similar to all muscles, PFMs require continuous exercise to remain strong once they are rehabilitated.

#### Behavioural interventions to improve outcomes of pharmacotherapy

Multicomponent behavioural training with urgency suppression techniques have been shown to enhance the effectiveness of pharmacotherapy for OAB (29). Thus, although antimuscarinics effectively inhibit detrusor contraction, patients may not be able to become continent without actively controlling urgency symptoms. For example, it has been reported that the addition of oxybutynin to a behavioural training regimen, and vice versa, yielded additional treatment benefit for older women with UUI who were not satisfied with the results of each therapy alone (84). Another study showed that the addition of multicomponent behavioural training to drug therapy reduced UUI episodes in women with OAB (85). However, this study also demonstrated that the behavioural training regimen did not increase the number of participants who could discontinue drug therapy while maintaining improvements in UUI, suggesting that behavioural training alone might not replace pharmacotherapy as a treatment for UI.

Evidence is accruing that behavioural interventions consisting of simple educational materials combined with verbal reinforcement from clinicians can improve OAB symptoms and increase patient satisfaction with pharmacotherapy. For example, combining a brief handout on bladder training and treatment with tolterodine was shown to significantly reduce the voiding frequency and increase the voided volume per micturition in participants with OAB compared with tolterodine treatment alone (*n* = 501) (45). Another study showed that significantly more participants receiving tolterodine in combination with an educational intervention either started or continued with drug treatment and reported an improvement in bladder symptoms, compared with those receiving tolterodine alone (*n* = 138) (86). A recent open-label study showed that combining tolterodine extended release with a simple self-administered behavioural intervention (Appendix 1) consisting of an educational pamphlet with monthly verbal reinforcement of the information resulted in high treatment satisfaction and improved OAB symptoms in participants who were dissatisfied with their most recent antimuscarinic OAB therapy (*n* = 416) (48). Participants in this study cited the information received on the causes of OAB and treatments available to improve bladder control as an important contributor to their treatment satisfaction (87). The results support the notion that many patients who are OAB pharmacotherapy ‘failures’ can become satisfied with the use of a tailored self-administered behavioural intervention. By contrast, a randomised parallel-group study reported that improvements in OAB symptoms and health-related quality of life elicited by darifenacin treatment were not enhanced by the addition of a self-administered behaviour modification programme (*n* = 395) (88). However, this study did not specify dissatisfaction with prior antimuscarinic treatment as an inclusion criterion, and the behavioural programme used multiple pamphlets and multimedia materials, contrasting with the focused written intervention used in the open-label tolterodine extended release study.

## Treatment pathways

Ideally, the treatment with the lowest risk should be recommended first in the treatment for OAB and UUI (89,90). Although behavioural interventions are associated with minimal risk, they can unfortunately be perceived as being time intensive and too difficult to implement in a busy office setting, especially when bladder symptoms become severe before they are recognised. Routine questioning of patients about voiding patterns would allow for symptoms to be recognised at an early point when information about healthy bladder habits could be optimally provided by the clinician. Screening for bladder symptoms is also important because of the negative impact that OAB symptoms and UI may have on other high priority conditions such as urinary tract infections, diabetes, obesity and hypertension. Scheduling a specific office visit to discuss bladder health issues and having the patient bring in a completed bladder diary would be an effective strategy in addressing any issues that might have been raised in a previous visit. The simple educational intervention described in this review (Appendix 1) could also enhance treatment outcomes for these other conditions and increase the value of the time invested by the clinician. This brief handout or similar material could easily be implemented by either a physician or a nurse. The key point is that the clinician invests the time educating patients about their condition and counsels them about strategies to manage their symptoms. It is important to keep in mind that treatment for other conditions and concomitant medical conditions may contribute to OAB symptoms, especially in older adults (91). Therefore, before adding another medication, it is important to ensure that an existing medication(s) is not contributing to the patient’s symptoms. If so, this should be taken into account when tailoring a behavioural intervention strategy.

In selecting the type of therapy to be used for the treatment of OAB and UUI, it is of paramount that clinicians gauge the patient’s treatment preferences, motivations, expectations and goals. The decision to begin behavioural interventions before or with medications is driven by clinician and patient preference. Some patients may want to experience quick treatment results and may not be receptive to behavioural intervention. In these cases, the optimal treatment option may be to prescribe an antimuscarinic in combination with education about lifestyle factors that can be changed to alleviate their OAB symptoms and UUI. Patients presenting with OAB symptoms should be taught skills for responding adaptively to urgency, including relaxation techniques, distraction techniques, PFM contractions and staying away from the bathroom until the urgency has subsided rather than rushing to the toilet. Bladder training or timed voiding can be especially helpful for patients whose symptoms include frequent urination. A guide to applying the techniques discussed in this review to the treatment of OAB symptoms and UUI is provided in Table 1. Additional information is available from the organizations listed in Appendix 3.

For the patient, the success of a lifestyle modification or behavioural intervention process, with or without pharmacological therapy, may depend on receiving adequate support from their healthcare provider. Clinicians should follow-up with patients regularly to monitor their progress and determine whether the treatment regimen is effective and satisfactory to the patient, or whether adjustments need to be implemented. If the patient is not responding satisfactorily to these first-line therapies, they can be referred to a continence specialist for evaluation and treatment. Urodynamic evaluation may be indicated for those with complex symptomatology. Patients who are having difficulty isolating their PFMs and desire behavioural intervention should be referred to a physical therapist, a continence nurse specialist, or a nurse practitioner for a more intensive behavioural intervention programme using biofeedback training and/or pelvic floor electrical stimulation. Biofeedback is a teaching technique that allows patients to monitor their PFM contractions and relaxations by providing immediate physiological feedback of PFM activity in an understandable visual and/or audible format (32). It has been shown that biofeedback-assisted behavioural training with PFMT in combination with education about strategies to control urgency reduced UI episodes by approximately 80%, which was greater than that observed for drug therapy alone in older women (44). Pelvic floor electrical stimulation involves the application of low-power electrical stimulation to the PFMs to induce a contraction of these muscles and a decrease in the uninhibited detrusor contractions associated with OAB (32).

## Conclusion

Behavioural interventions can readily be incorporated into the daily lives of patients who possess the cognitive and functional capability; clinicians and staff in busy office settings can readily incorporate these therapies into routine medical and nursing care. Although OAB symptoms and UUI can be successfully managed using non-pharmacological approaches, they require considerable motivation from the patient and attrition rates may be high without adequate follow up, although attrition may also occur because of lack of efficacy. Behavioural interventions which educate and empower patients can be utilised either alone or as an adjunct therapy to enhance pharmacotherapy for OAB and UUI. However, the issue of whether it is optimal to initiate treatment using a combined therapy or to start with a single therapy and then introduce a second approach if the patient does not respond to treatment remains to be resolved and may be largely driven by patient preference until more evidence is available. Finally, clinicians should be familiar with the practical details of habit changes and training techniques not only to optimise treatment outcomes in consideration of patient preferences and goals for OAB treatment, but also as the foundation for patient education to promote bladder health as part of routine healthcare.

## Figures and Tables

**Appendix 1:**
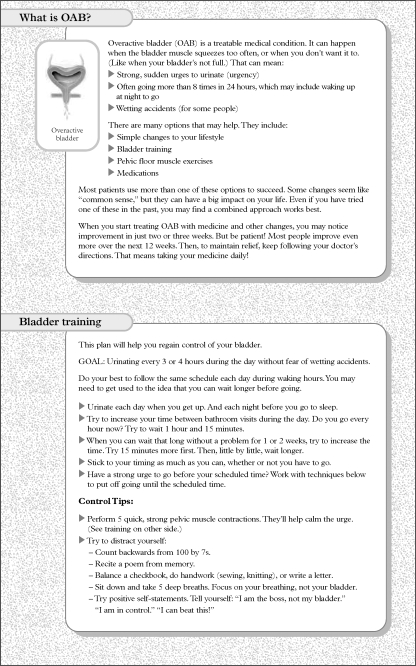

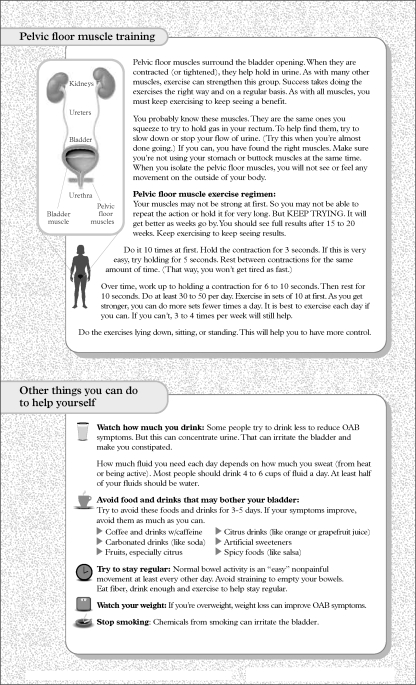
Educational handout. Male and female versions of the OAB patient education sheet are available at http://www.pfizerpro.com/toviaz in the patient education section

## Appendix 2

**Table:** Daily voiding record

		Amount of urine leakage			Changed wet pad			
Time interval	Urinated in toilet	□L	□M	□S	Reason for urine leakage	□D	□W	□S	Type/amount of liquid intake
Sample	√	□L	√M	□S	rushing to toilet	□D	□W	√S	
6 am		□L	□M	□S		□D	□W	□S	
7 am		□L	□M	□S		□D	□W	□S	
8 am		□L	□M	□S		□D	□W	□S	
9 am		□L	□M	□S		□D	□W	□S	
10 am		□L	□M	□S		□D	□W	□S	
11 am		□L	□M	□S		□D	□W	□S	
Noon		□L	□M	□S		□D	□W	□S	
1 pm		□L	□M	□S		□D	□W	□S	
2 pm		□L	□M	□S		□D	□W	□S	
3 pm		□L	□M	□S		□D	□W	□S	
4 pm		□L	□M	□S		□D	□W	□S	
5 pm		□L	□M	□S		□D	□W	□S	
6 pm		□L	□M	□S		□D	□W	□S	
7 pm		□L	□M	□S		□D	□W	□S	
8 pm		□L	□M	□S		□D	□W	□S	
9 pm		□L	□M	□S		□D	□W	□S	
10 pm– Midnight		□L	□M	□S		□D	□W	□S	
Midnight – 2 am		□L	□M	□S		□D	□W	□S	
2–4 am		□L	□M	□S		□D	□W	□S	
4–6 am		□L	□M	□S		□D	□W	□S	

© Diane K. Newman.

### Directions

Column 1: Time interval.

Column 2: Next to the correct time interval, place a checkmark (√) next to time urinated.

Column 3: Mark every time you have urine leakage and indicate whether the amount was large (L), moderate (M) or small (S).

Column 4: Record the reason for the urine leakage, such as sneezing, lifting, coughing, laughing, could not make it to the bathroom, and so on.

Column 5: Place a checkmark (√) each time a wet pad was changed. Mark how wet: ‘D’ if the pad is slightly wet or damp, ‘W’ if wet, and ‘S’ if the pad is saturated or very wet.

Column 6: In the correct time interval, describe your liquid intake (e.g. coffee, water and orange juice) and estimate the amount (e.g. one cup or 8 oz).

## Appendix 3

**Table:** Resources

Alliance for Ageing Research	2021 K St. NW, Suite 305 Washington, DC 20006 (202)293-2856 http://www.agingresearch.org
American College of Obstetricians and Gynecology (ACOG)	409 12th, SW Washington, DC 20024 (202) 638-5577 http://www.acog.com
American Urological Association Foundation (AUAF)	1000 Corporate Boulevard Linthicum, MD 21090 (866) 746-4282 http://www.auafoundation.org/
American Physical Therapy Association	Section on Women’s Health 111 N. Fairfax St. Alexandria, VA 22314 (703) 684-2782 http://www.apta.org
American Urogynecologic Society (AUGS)	2025 M St. NW, Suite 800 Washington, DC 20036 (202) 367-1167 http://www.augs.org
American Urological Association (AUA)	1120 N. Charles St. Baltimore, MD 21201 (410) 727-1100 http://www.auanet.org
Association of Women’s Health, Obstetric and Neonatal Nurses	2000 L St. NW, Suite 740 Washington, DC 20036 (800) 673-8499 http://www.awhonn.org
International Continence Association	19 Bristol Square Bristol Bristol BS2 8SJ UK (44) 117 944881 http://www.icsoffice.org
National Association for Continence (NAFC)	PO Box 1019 385 Meeting St., Suite 100 Charleston, SC 29402 (800) 252-3337 http://www.nafc.org
National Institute of Diabetes and Digestive and Kidney Disease (NIDDK)	National Institutes of Health Westwood Building, Suite 3A-05 Bethesda, MD 20892 http://www.niddk.nih.gov
Simon Foundation for Continence	Box 835-F Wilmette, IL 60092 (800) 237-4666 http://www.simonfoundation.com
Society of Urologic Nurses and Associates (SUNA)	East Holy Avenue, Box 56 Pitman, NJ 09071-0056 (888) 827-7862 http://www.suna.org
Wound, Ostomy and Continence Nurses Society (WOCN)	4700 West Lake Avenue Glenview, IL 60025 (800) 224-9626 http://www.wocn.org

## Supporting information

**Appendix S1**. Bladder record
