# Supplementary material for: Practical aspects of lifestyle modifications and behavioural interventions in the treatment of overactive bladder and urgency urinary incontinence
Source: Int J Clin Pract. 2009 Aug;63(8):1177–91. doi: 10.1111/j.1742-1241.2009.02078.x (PMC2734927; doi:10.1111/j.1742-1241.2009.02078.x)
Supplement: Supplementary file 1 [file ijcp0063-1177-SD1.doc]

# BLADDER RECORD

# Name:_______________________________________ Date: ____________________

## INSTRUCTIONS

# Column 1 Times are noted in 2 hours periods.

Column 2 Measure and record the amount of urine voided.

Column 3 Note when you leaked urine and these codes to indicate the amount of urine.

**S = Slightly damp;**

**M = Pad or underwear definitely wet, at least a tablespoon;**

**L = Wet outer garments, large urine loss.**

Column 4 Indicate with an “X” if you felt urgency or the need to void at the time of leakage

Column 5 Describe the activity you were performing at the time of leakage of urine (examples are sneezing, coughing, lifting, “couldn’t make it to the bathroom.”

Column 6 Describe the type of liquid (fluids) you drink throughout the day (i.e., coffee, water, etc.) and estimate of the amount (i.e., one cup, large 8 oz glass).

| **Column 1** | **Column 2** | **Column 3** | **Column 4** | **Column 5** | **Column 6** |
| --- | --- | --- | --- | --- | --- |
| **Time** | **Voided (X) in Toilet with Voided Amount** | **Urine Leakage**  **S M L** | **Urge Present** | **Activity with Leakage** | **Liquid Intake** |
| 6 – 8 a.m. |  |  |  |  |  |
| 8 – 10 a.m. |  |  |  |  |  |
| 10 am – Noon |  |  |  |  |  |
| Noon – 2 p.m. |  |  |  |  |  |
| 2 – 4 p.m. |  |  |  |  |  |
| 4 – 6 p.m. |  |  |  |  |  |
| 6 – 8 p.m. |  |  |  |  |  |
| 8 – 10 p.m. |  |  |  |  |  |
| 10 – 12 Mid |  |  |  |  |  |
| 12 Mid –2 a.m. |  |  |  |  |  |
| 2 – 4 a.m. |  |  |  |  |  |
| 4 – 6 a.m. |  |  |  |  |  |

Please write the number of absorbent pads (protective garments) you used this day, the type and name of the products.

# Number of Pads used today: _____________ Type and/or name: ___________________

ANY COMMENTS? ______________________________________________________

**©Diane K. Newman**
